# Fiber Quality Improvement in Upland Cotton (*Gossypium hirsutum* L.): Quantitative Trait Loci Mapping and Marker Assisted Selection Application

**DOI:** 10.3389/fpls.2019.01585

**Published:** 2019-12-11

**Authors:** Babar Ijaz, Nan Zhao, Jie Kong, Jinping Hua

**Affiliations:** ^1^Laboratory of Cotton Genetics, Genomics and Breeding/Key Laboratory of Crop Heterosis and Utilization of Ministry of Education/Beijing Key Laboratory of Crop Genetic Improvement, College of Agronomy and Biotechnology, China Agricultural University, Beijing, China; ^2^Institute of Economic Crops, Xinjiang Academy of Agricultural Sciences, Urumqi, China

**Keywords:** fiber quality, genetic mechanism, molecular markers, QTL mapping, fine mapping, *Gossypium* species

## Abstract

Genetic improvement in fiber quality is one of the main challenges for cotton breeders. Fiber quality traits are controlled by multiple genes and are classified as complex quantitative traits, with a negative relationship with yield potential, so the genetic gain is low in traditional genetic improvement by phenotypic selection. The availability of *Gossypium* genomic sequences facilitates the development of high-throughput molecular markers, quantitative trait loci (QTL) fine mapping and gene identification, which helps us to validate candidate genes and to use marker assisted selection (MAS) on fiber quality in breeding programs. Based on developments of high density linkage maps, QTLs fine mapping, marker selection and omics, we have performed trait dissection on fiber quality traits in diverse populations of upland cotton. QTL mapping combined with multi-omics approaches such as, RNA sequencing datasets to identify differentially expressed genes have benefited the improvement of fiber quality. In this review, we discuss the application of molecular markers, QTL mapping and MAS for fiber quality improvement in upland cotton.

## Introduction

Among 53 *Gossypium* species, there are four cultivated species, including *Gossypium Hirsutum*, *G. Barbadense*, *G. Arboreum*, and *G. Herbaceum* (Fryxell, 1992; Wendel and Grover, 2015; Gallagher et al., 2017; Wang et al., 2018). Upland Cotton (*G. Hirsutum* L.) is extensively cultivated due to its wide adaptability to the environment, high production, and better yield potential, which fulfils over 95% of the output of global cotton yield (Chen et al., 2007). Sea Island cotton (*G. Barbadense*) is known for excellent fiber quality with long, strong, and fine fibers (Avci et al., 2013). To meet the demands of modern textile industry, genetic improvements in cotton have been performed in fiber quality and yield traits in different populations along with cotton production. Therefore, it is a great challenge to improve the fiber quality and to increase the yield potential simultaneously in diverse areas and planting systems in cotton production.

Fiber quality traits generally include fiber length, fiber uniformity, fiber strength, fiber elongation, and micronaire value. Fiber strength and fiber length are considered as the most important properties affecting yarn quality (Yang, et al., 2016). Fiber strength is very important for advanced spinning technologies in the textile industry (Felker, 2001). The micronaire value, a measure of fiber fineness and fiber maturity, influences the fiber processing and dyeing consistency (Rodgers et al., 2017).

**Figure 1:**
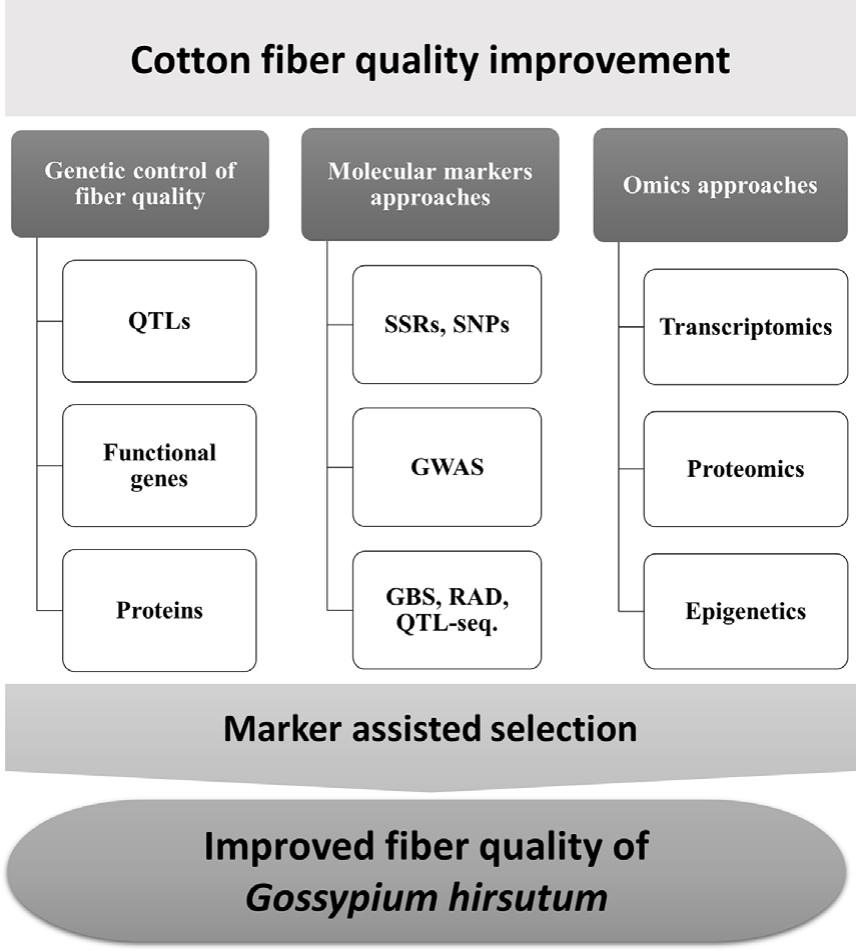
Overview of the strategies for marker-assisted selection (MAS) to improve fiber quality in *Gossypium hirsutum*.

Although studies in fiber development have been engaged for many years, the molecular mechanism is still not well explained (Zhang et al., 2013). Quantitative trait loci (QTL) mapping with molecular markers provides a powerful approach to dissect the molecular mechanism underlying complex fiber quality traits. At least 1075 QTLs from 58 studies and 1059 QTLs within 30 studies have been reported with intraspecific and interspecific crosses (Said et al., 2015b), which provide a platform for follow-up QTL validation and gene cloning in dissecting the mechanism involving complex traits. Functional genomic studies provide new insights to better understand fiber development mechanisms in *Gossypium* species (**Figure 1**). Here, we endeavor to provide comprehensive information on QTL mapping and utilization in cotton.

## Overview of Fiber Development Mechanism in Cotton

Cotton fiber is a seed-borne epidermal trichome composed of a highly elongated and thickened single cell. Cotton fiber achieves the expansion through water influx with changes of turgor pressure in a cell (Cosgrove, 1997). Generally, the fiber cell develops into a trichome in four stages: a) fiber initiation, b) cell elongation [primary cell wall (PCW) synthesis], c) cell wall biosynthesis [secondary cell wall (SCW) deposition], and d) fiber maturation (Kim and Triplet, 2001; Ruan, 2005; Pang et al., 2010; Zhang et al., 2013; Zhang and Liu, 2013). The fiber initiation starts from 3 days before anthesis to 3 days post anthesis (DPA) with trichome protrusion and enlargement of epidermal cells (Qin and Zhu, 2011). In some *G. hirsutum* cultivars, lint percentage and lint index are positively correlated with the density of 0 DPA (fiber protrusion) and 1 DPA fiber initials (fiber elongation; Li et al., 2009).

Fiber elongation starts from 2 DPA to 20 DPA (typically) after the initiation period, these elongating fibers twist together to develop into bundles of fiber (Singh et al., 2009; Haigler et al., 2012). The closure of plasmodesmata is important in fiber cell elongation with the increase of cellulose synthesis at 10-16 DPA (Ruan et al., 2004). At a cellular level, several mechanisms facilitate the rapid cotton fiber elongation; as an example, *GhVIN1* expression increased in *G. barbadense* at a higher level until 10 DPA during fiber elongation (Wang et al., 2010). Aquaporin proteins also facilitate cotton fiber elongation and the corresponding genes, including *GhγTIP1* and *GhTIP1-2* in the tonoplast and plasma membrane, respectively (Liu et al., 2008). The expression of pectin biosynthesis genes promotes fiber elongation by ethylene signaling pathways (Qin et al., 2007; Pang et al., 2010). Expansins are likely to play a role in regulating fiber elongation (Li et al., 2016). Dynamic DNA methylation has been reported during cotton fiber differentiation, and additionally, lncRNAs are associated with chromatin modifications (Wang et al., 2015a; Wang et al., 2016a).

During the transition phase of cell elongation to cell wall biosynthesis, fiber elongation stops gradually and SCW is thickened with the biosynthesis of SCW genes up-regulated (Hinchliffe et al., 2010). SCW thickening depends upon the increased rate of cellulose synthesis, orientation of cellulose fibrils, and greater length of individual cellulose chains (Haigler et al., 2009; Betancur et al., 2010). Cellulose fibrils in primary and secondary walls are synthesized by cellulose synthases (CesAs) and cellulose synthase-like enzymes, which initiates cell wall matrix polymers (Taylor et al., 2003). Several flavonoid and lignin biosynthesis pathways regulate the start of SCW synthesis in sclerenchyma cells (Ehlting et al., 2005; Zhao and Dixon, 2011). Single-cell expression systems will provide a better understanding for the molecular control of cotton fiber elongation dynamics and cell wall biosynthesis in the future. Genes controlling cellulose synthesis of cotton fiber are orthologs of other vascular plants (Haigler et al., 2005; Haigler et al., 2009). The PCW features of *Arabidopsis* leaf trichomes are integrated with SCW synthesis of cotton the fiber during cell wall thickening (Betancur et al., 2010), which offers a strategy and technology roadmap to validate functions of related genes.

## The Underlying Genomic and Proteomic Modifications in Control of Fiber Development

### Overview of the Genes Involved in Fiber Development

Transcriptome profiles reveal novel information in different stages of fiber development. A number of transcription factor (TF) families, for example, MYB, WRKY, AP2/EREBP, C2H2, and bHLH, were involved in fiber initiation (**Table 1**; Yang et al., 2006). The diverse TF genes in different *Gossypium* species (*GbML1, GaMYB2*, *GhMYB109*, and *GaHOX1*) are expressed differentially during fiber initiation and the early stage of fiber development (Wang et al., 2004; Guan et al., 2008; Pu et al., 2008; Zhang et al., 2010).

**Table 1:** Some potential genes involved in cotton fiber development.

Gene	Accession no.	Protein	Potential function	Reference
14-3-3L	DQ402076	14-3-3l	Expressed at early stages of fiber development, involved in regulation of fiber elongation.	Shi et al., 2007
CAP	AB014884	Adenylyl cyclase associated protein	Highly expressed in young fibers and play an important functional role in early stages of fiber development.	Kawai et al., 1998
CEL	AY574906	Endo 1,4-β-glucanase	Necessary for plant cellulose biosynthesis and expressed during secondary cell wall synthesis	Pear et al., 1996
CelA1	GHU58283	Cellulose synthase	Expressed in secondary cell wall synthesis, involved in synthesis of cellulose	Chen and Burke 2015
CelA3	AF150630	Cellulose synthase catalytic subunit	Involved in cellulose biosynthesis stage in developing cotton fibers	Zhang et al., 2012
CIPK1	EF363689	CBL-interacting protein kinase	Expressed at elongation phase when developing fiber	Gao et al., 2007
Exp1	DQ204495	α-Expansin 1	Cell wall extension in fiber development and influence length and quality of fiber	Shi et al., 2006
Exp	DQ060250	Expansin	Modify the mechanical properties of cell wall, affect the length and quality of cotton fibers by enabling turgor-driven cell extension.	Zhu et al., 2012
ACT1	AY305723	Actin 1	Expressed during fiber development and participate in fiber elongation	Li et al., 2005
BG	DQ103699	β-1,4-Glucanase	Play its role in loosening of primary cell wall and promotion of secondary cell wall synthesis.	Ma et al., 2006
ManA2	AY187062	β-Mannosidase,	Glycosyl hydrolase and expressed during fiber development	Zhu et al., 2011
Pel	DQ073046	Pectate lyase	Degrade the de-estrified pectin and has a role in process of fiber elongation in cotton	Bai et al., 2014
RacA	DQ667981	Small GTPase	Expressed during fiber elongation, might play a role in early stage of fiber development	Li et al., 2005a
RacB	DQ315791	Small GTPase	Expressed during secondary cell wall thickening, may have role in fiber quality	
Sus1	U73588	Sucrose synthase	Expressed in secondary cell wall thickening, plays a role in fiber initiation and elongation by influencing carbon partitioning to cellulose synthesis.	Ruan et al., 2003
LTP3	AF228333	Lipid transfer protein gene	Involved in Cutin synthesis during fiber primary cell wall synthesis	Liu et al., 2000
SusA1	HQ702185	Sucrose synthase	Potentially play an important role in the elongation of cotton fiber process	Jiang et al., 2011
HOX3	107904747	Homeodomain protein	Controls fiber elongation and associated with quantitative trait loci (QTLs) for fibre length.	Wang et al., 2004
CAM7	TC232366	calmodulin protein	Promotes cotton fiber elongation by modulating reactive oxygen species (ROS) production	Cheng et al., 2016

The transcriptional expression of *GhHD1*, *GhMYB*-25, *GhSusA1*, *GhFLA1*, *GbPDF1*, and *GhVIN1* genes contributes to the initial stage of fiber development (Machado et al., 2009; Walford et al., 2011; Deng et al., 2012; Jiang et al., 2012; Huang et al., 2013; Wang et al., 2014), in which *GhFLA1*, an arabinogalactan protein gene, enhanced fiber initiation and fiber elongation with the alteration of PCW integrity (Huang et al., 2013). The *Myb2* TF triggered the expression of R22-like (RDL) gene in the stage of fiber initiation (Suo et al., 2003; Wang et al., 2004). The silencing of the cotton *GbPDF1* (PROTODERMAL FACTOR1) gene retarded fiber initiation and produced shorter fibers compared with wild type (Deng et al., 2012).

Cellulose synthase catalytic subunits (CesAs) regulated the cellulose synthesis pathways by catalytic sites within a multi-subunit complex, at least 15, 14, and 29 CesA genes have been reported in *G. raimondii*, *G. arboreum*, and *G. barbadense*, respectively (Paterson et al., 2012). Moreover, the up-regulation of CESa genes (*GhCES5*–*GhCESA10* in *G. hirsutum*; CesA4, CesA7 and CesA8 in *G. barbadense*) were involved in primary and SCW biosynthesis (Li et al., 2013; Liu, et al., 2015; Nawaz et al., 2017), post-translational modifications, and trafficking (Polko and Kieber 2019).

Plant hormones control fiber cells development by regulating gene expression at different growth levels. The roles of brassinosteroid (BR), jasmonic acid (JA), auxin, and arabinogalactan protein (AGP) biosynthetic pathways have been revealed to be involved in up-regulating or suppressing the expression of genes in both fiber initiation and fiber elongation (Yang et al., 2014; Zhang et al., 2016; Qin et al., 2017). *GhPIN*-mediated auxin transport was responsible for ovule-specific suppression of the *GhPIN* gene, demonstrating its roles in fiber initiation *via* the auxin accumulation (Zhang et al., 2016). DELLA-like proteins acted as repressors in gibberellic acid (GA) signaling pathway, for example, four genes (*GhGAI3a*, *GhGAI3b*, *GhGAI4a*, and *GhGAI4B*) containing DELLA domains expressed during fiber initiation and elongation (Wen et al., 2012). *GhCaM7* played an important part in the initiation and elongation stages of fiber cells by controlling the production of reactive oxygen species and H_2_O_2_ which regulated Ca^2+^ influx into the fiber (Tang et al., 2014).

*ACTIN1*, *GhPIP2*, *GhCaM7*, *WLIM1a*, *GhPAG1*, and *GhHOX3* played important roles in the fiber elongation process (Li et al., 2005; Han et al., 2013; Li et al., 2013; Shan et al., 2014; Tang et al., 2014; Yang et al., 2014). The *GhBZR1* protein regulated fiber elongation by binding to the promoters of genes *GhXTH1* and *GhEXP* (Zhou et al., 2015). Expansin proteins play critical roles in cell wall loosening and fiber elongation by producing polysaccharide complexes including xyloglucan and pectin, with the 3-year field performance of *GhEXPA8* transgenic plants showed longer fiber and better micronaire (Bajwa et al., 2015). *PAG1* gene controlled endogenous BRs level by encoding a cytochrome P450 and played an important role in regulating fiber elongation (Yang et al., 2014). *GhMYB212* regulates the expression of *GhSWEET12* during cell expansion at developing phases of fiber elongation (Sun et al., 2019). Further studies involved in gene function elucidation will enhance the understanding in the mechanism underlying fiber development.

### The Major Proteins Involved in Cotton Fiber Development

The availability of cotton genome sequences makes post-genomic proteomic studies possible, which further helps to provide new understandings into cotton fiber initiation and elongation mechanisms. All the genes function by accomplishing protein modifications in different development stages of cotton fiber. Many proteins, acting as enzymes, make contributions in the basic energy metabolism pathways, such as polysaccharide biosynthesis, glycolytic pathway, the pentose-phosphate, and the tricarboxylic acid cycle (Yang et al., 2008; Pang et al., 2010; Liu et al., 2012; Du et al., 2013; Li et al., 2013a; Zhang and Liu, 2013). Some proteins are also involved in hormone signaling pathways, such as GA, jasmonate (JA), BR, ethylene (ET) and abscisic acid (ABA) (Pang et al., 2010; Liu et al., 2012; Du et al., 2013; Zhang et al., 2013).

Proteomic analyses showed the involvement of redox homeostasis-related proteins, including dehydroascorbate reductase (DHAR), ascorbate peroxidase (APX), catalase (CAT), nicotineamide adenine dinucleotide phosphate-isocitrate dehydrogenase (NADP-ICDH), and phospholipase D alpha (PLDα), in cotton fiber elongation (Yang et al., 2008). H_2_O_2_ acted as a signaling element involved in controlling the growth and development of plants, that is, the lower the level of H_2_O_2_ was associated with the higher levels of DHAR, CAT at 5 DPA and 10 DPA, respectively, showing the H_2_O_2_ producing and scavenging systems in elongating fiber cells (Mittler et al., 2004; Du et al., 2013).

Cytosolic pyruvate kinase involved in catalyzing the glycolytic pathway was highly expressed at 20 DPA, suggesting its role in the process of fiber elongation to secondary wall thickness (Duggleby and Dennis, 1973; Zhang et al., 2015). Some enzymes (such as transketolase) catalyze several reactions in glycolysis and were up-regulated in cotton fiber elongation, implying the key role of carbohydrate metabolism in fiber development at transcriptional level (Zhang et al., 2015). Similarly, the expression of the *Sus* gene was higher at early stages and lower at later stages of fiber elongation because sucrose was converted into fructose and UDP-glucose in cell expansion and cell wall biosynthesis (Delmer, 1987; Ruan et al., 2003). Furthermore, the transition phase from PCW to SCW synthesis was accompanied with the biosynthesis of guanosine diphosphate fucose, suggesting the roles of more fucosylation (Freshour et al., 2003). The proteomic profiles of fuzzless-lintless (*fl*) mutant and wild-type upland cotton fiber revealed that pectin synthesis was necessary for fiber elongation (Pang et al., 2010), supporting the hypothesis that fiber elongation was regulated through redox homeostasis by the evidence that proteins involved in the cellular redox homeostasis were expressed differentially in *fl* mutant (Liu et al., 2012).

Proteomic approaches in cotton fiber domestication and evolution provide databases for functional genomics analysis in cotton fiber. In total, 190 differentially expressed proteins were identified between wild and cultivated accessions using iTRAQ technology (Hu et al., 2013). Proteome comparison between *G. hirsutum* and *G. barbadense* with their diploid progenitors revealed that the two allopolyploid species have attained superficially ‘modern’ fiber phenotypes during evolution (Hu et al., 2015). However, the early events in fiber elongation have been neglected so far and could be characterized at the proteome level.

## Molecular Markers and Linkage Maps for Fiber Quality Traits

The applications of molecular markers allow the development of high density genetic linkage map and germplasm evaluation, as well as phylogenetic and evolutionary analysis. Desired alleles and high density linkage maps will be helpful for mapping of stable QTLs and efficient gene identification controlling good quality fiber. Molecular markers including random amplified polymorphic DNA, restriction fragment length polymorphism, simple sequence repeats (SSRs), inter simple sequence repeats, and single nucleotide polymorphisms (SNPs) (Tatineni et al., 1996; Liu et al., 2000; Abdalla et al., 2001; Lu and Myers 2002; Reddy et al., 2002; Zhu et al., 2003; Alvarez and Wendel, 2006; Li et al., 2015) have been used for QTL identification for fiber quality traits (Said et al., 2015b). Genetic maps have also been proven to be useful to uncover the molecular bases of multi-genic traits such as fiber quality. Mapping populations between diverse sets of cultivars from distinct geographic origins were used to determine the positive associations between genetic markers and fiber traits. Several major QTLs that are persistent among the populations show high phenotypic variation (PV) and likelihood of odds score. The first genetic map of cotton that covered a length of 4675 cM with 705 restriction fragment length polymorphism markers was developed by Reinisch et al. (1994). Afterward, different genetic maps have been developed and identify 14, 26 and 13 QTLs for fiber quality (Jiang et al., 1998; Ulloa et al., 2000; Kohel et al., 2001), 28 QTLs for fiber length, 9 QTLs for fiber length uniformity, and 8 QTLs for short fiber index (Chee et al., 2005).

### SSR and SNP Markers for Fiber Quality Traits

With the availability of cotton genome sequences and the development of bioinformatics tools, the rapid increase of SSR markers and SNPs has become more effective for genotyping and QTL mapping in cotton. SSRs have been widely utilized for genetic dissection of QTL mapping in different wild and domesticated cotton species. There are a number of studies conducted to identify fiber quality related QTLs using SSR genetic maps with the use of intraspecific populations and under multiple environments and generations (Zhang et al., 2003; Lacape et al., 2005; Zhang et al., 2005; Wang et al., 2006; Wang et al., 2007; Zhang et al., 2009; Sun et al., 2012; Liang et al., 2013; Fang et al., 2014; Zhiyuan et al., 2014; Shao et al., 2014;Ma et al., 2019). Shen et al. (2005) performed extensive SSR genotyping using 1378 markers in an F_2_ population and detected 39 QTLs linked with fiber quality traits among them, 11 QTLs for fiber length, 10 for fiber strength, 9 for micronaire, and 9 for fiber elongation.

A significant association had been found between SSRs and fiber quality traits in *Gossypium* species (Zeng et al., 2009). Association mapping of fiber quality traits linked with SSR markers identified 52.86% (70 stable loci) for target traits including 30 for fiber length, 27 for fiber strength, and 13 for fiber fineness (Cai et al., 2014). Wang et al. (2015) constructed a genetic linkage map containing 644 polymorphic loci and identified a total of 64 QTLs (10 QTLs for fiber length, 18 for fiber strength, 13 for micronaire, 16 for fiber elongation, and 7 for fiber length uniformity ratio) associated with fiber quality in seven environments. Another linkage map comprising of 579 markers was developed using SSRs, conserved intron scanning primers, and transcript-derived fragment amplifications, with an average distance of 7.19 cM between markers, in a backcross population (BC) derived from a cross among *G. hirsutum* and *G. barbadense* (Yang et al., 2015). Nie et al. (2016) used 494 genome-wide SSR markers and 503 indigenous upland cotton lines to map 216 marker loci linked with fiber quality traits (61 loci with fiber strength, 46 for fiber upper half mean length, 25 for fiber elongation, 23 for micronaire value, 19 for fiber uniformity, and 42 for short fiber) and some novel alleles that could be considered as selection tags for breeding programs.

Shang et al. (2016) explored fiber quality in two recombinant inbred line (RIL) populations along-with their corresponding BC populations and identified 62 common QTLs in multiple environments and populations; 286 QTLs were also detected by the digenic interactions and their environmental interactions (QEs) analyses under multiple environments; such results concluded that single-locus and epistasis along with minor measureable major effects showed an important role in regulating fiber quality in upland cotton. Ma et al. (2017) developed a BC population from a RIL population and compared the genetic mapping effects on RIL and BC populations. The 26 and 37 QTLs were detected for fiber quality in RIL and BC populations, respectively, yet there was no significant role of heterosis observed in fiber quality traits (Ma et al., 2017). Li et al. (2018) utilized immortalized F_2_ population with two reciprocal BC populations and identified total 167 QTLs, whereas, multi-environment analysis offered 104 main-QTLs and 114 epistasis-QTLs in both populations referred over-dominance and epistatic factors are important to fiber quality heterosis in cotton. In total, 134 SSR marker-trait associations were identified for four fiber quality traits; of these, 15 significant associations explained 12%–16% of total phenotypic variance for the markers NAU5120, DPL0378, and JESPR101 (Adhikari et al., 2017). A total of 68 QTLs including 11 stable QTLs for five fiber quality traits were detected in RIL by SSR map containing 2051 loci (Liu et al., 2017). Huang et al. (2018) applied 284 SSR markers to identified 14 stable QTLs linked with fiber quality traits in a multi-parent advanced generation inter-cross population with 960 lines developed from eight parents. Similarly, three sets of introgression line (ILs) populations developed from *G. hirsutum* race stocks (TX-34, TX-48, and TX-114) were genotyped with 452 SSR markers, and identified 38 new QTLs related to fiber quality traits, among them 17 favorable QTLs demonstrate the importance of race stock germplasm in cotton fiber improvement (Feng et al., 2019).

### High-Throughput Sequencing Techniques for Fiber Quality Traits

The availability of genomic sequences of the *G. raimondii* (D_5_), *G. arboreum* (A_2_), *G. hirsutum* (AD_1_), and *G. barbadense* (AD_2_) provides more opportunities to improve the density of intraspecific genetic maps (Paterson et al., 2012; Wang et al., 2012; Li et al., 2014; Li et al., 2015; Liu et al., 2015; Yuan et al., 2015; Zhang et al., 2015). Soon after the genome sequences in *Gossypium* became available, molecular markers were used to develop high precision QTL linkage maps. High-throughput genotyping offers a deep insight into genome wide genetic maps for a wide range of applications in QTL mapping and marker assisted selection. For example, the development of CottonSNP63K (Hulse-Kemp et al., 2015) and CottonSNP80K (Cai et al., 2017) arrays has allowed genetic mapping, genomic selection, genome-wide association studies, and an understanding of genomic diversity among *Gossypium* breeding populations. Common QTLs related to fiber quality in different environments identified through SNP markers can be deployed as a priority for fine mapping and candidate genes identification (Li et al., 2016; Su et al., 2016; Tan et al., 2018).

The advance techniques in next generation sequencing have boosted the genetic maps quality and validity with SNP polymorphisms to increase marker coverage across the genome. These technologies include restriction-site associated DNA (Wang et al., 2015; Jia et al., 2016), specific locus amplified fragment sequencing (SLAF-seq) (Zhang et al., 2016), and genotyping by sequencing (Qi et al., 2017). Zhang et al. (2019) performed GWAS analysis with the combined association and linkage mapping approach and identified two potential QTLs on chromosome D03 and D08 (qFL-D03-1/2 and qFL-D08-1/2) in multiple environments. Further identification of candidate genes reveals 26 genes on chromosome D03 and among them, the Gh_D03G1338 was potentially involved in the fiber development stages (Zhang et al., 2019). Another study, performed multi-locus GWAS and genotyping was done by the SLAF-seq approach, identifying several quantitative traits nucleotides (QTNs), D11_21619830, A05_28352019, and D03_34920546 for fiber quality traits and detected six genes highly expressed in cotton fibers (Su et al., 2018). Li et al. (2018) conducted both single locus and multi-locus GWAS and identified collectively 342 QTNs for fiber quality traits, and nine QTNs with maximum PV. A multi-parent advanced generation inter-cross population with 550 individuals were utilized for GWAS analysis combined with RNA-seq of lines with extreme fiber characteristics, identified highly significant QTL on D11 which further dissected into 12 genes and an auxin-responsive GH3 gene (Gh_D11G1989) as the candidate gene for fiber length (Naoumkina et al., 2019). Fan et al. (2018) constructed another high-density map with a *G. barbadense* (5917 and American Pima S-7) RIL population locating 42 QTLs for fiber quality traits, distributed among 14 linkage groups, with the number of QTLs located in A subgenome was 28 whereas only 14 QTLs were associated with D subgenome. An intraspecific population of *G. hirsutum* was utilized for genotyping by sequencing mapping and 30 consistent QTLs were identified from 110 common QTLs, divided into two main clusters on chromosome D03 and D12 (Diouf et al., 2018). Five candidate genes (Gh_D03G0889, Gh_D12G0093, Gh_D12G0410, Gh_D12G0435, and Gh_D12G0969) were associated with QTLs (qFM-D03_cb, qFS-D12_cb, and qFY-D12_cb), functions in protein kinases and phosphorylation, respectively (Diouf et al., 2018).

## Distribution and Resources of QTL Mapping for Fiber Quality Traits

**Figure 2:**
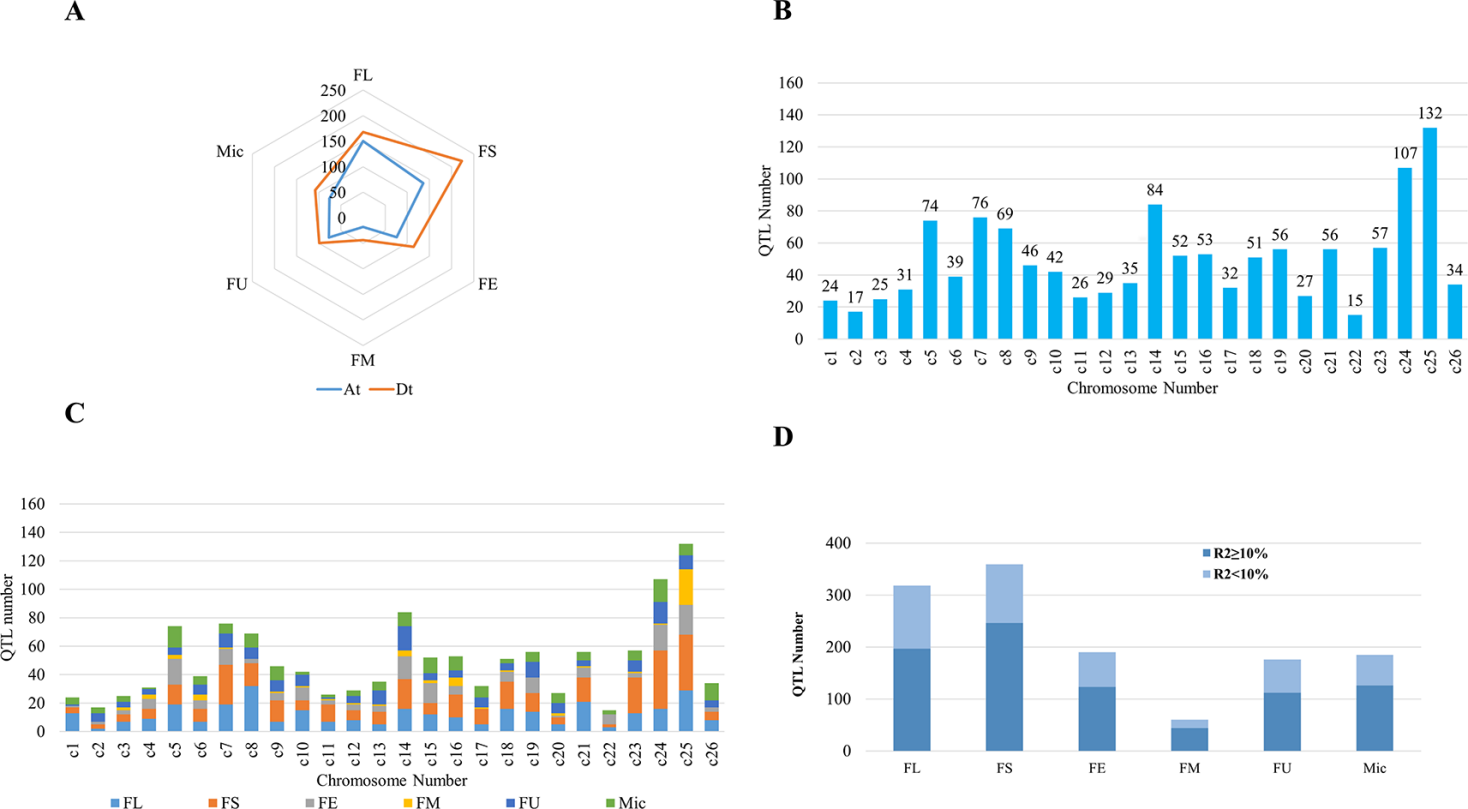
Quantitative trait loci (QTLs) distributed across each chromosome in cotton genome. **(A)** QTLs of fiber quality traits distributed across At and Dt sub-genomes. **(B)** QTLs mapped in each chromosome. c1–c26 represents chromosome 1 to chromosome 26, respectively. **(C)** QTLs for different fiber quality traits. FL, fiber length; FS, fiber strength; FE, fiber elongation; FM, fiber maturity; FU, fiber uniformity; Mic, micronaire. **(D)** The variance (R2) explained by a single QTL related to fiber quality traits. FL, FS, FE, FM, FU, Mic. The same as used above.

A number of QTLs associated with fiber quality traits have been identified in different mapping populations such as RILs, bi-parental segregating populations, and BC populations within intraspecific populations among *G. hirsutum* and interspecific crosses of *G. hirsutum* and *G. barbadense*. In particular, nearly 80% of fiber quality QTLs were identified from the interspecific populations were due to the wide genetic base (https://www.cottongen.org/; Yu et al., 2014). To date, more than 1500 QTLs for fiber traits have been mapped (Said et al. 2015a;Yang et al., 2015), providing the potential to be manipulated by MAS for the improvement of cotton fiber length and strength. Overall, for six fiber quality traits (FL, FS, FE, FM, FU, and Mic), At sub-genome linked with more number of QTLs than Dt sub-genome (**Figure 2A**). The number of QTLs for different fiber quality traits also varies over the chromosomes, with the maximum number of 359 QTLs distributed across the genome for fiber strength followed by fiber length with 318 QTLs spread over 26 chromosomes (**Figure 2B**). Whereas, the fiber maturity had minimum number of 62 QTLs in cotton genome. According to Said et al. (2015b), majority of the QTLs were located around the first 0–20 cM of each chromosome with few exceptions, and this seems to be evolutionarily conserved.

The number of fiber quality trait QTLs over the chromosomes of the cotton genome is not identical, some chromosomes containing more QTLs than the others. QTLs associated with cotton fiber quality obtained from CottonQTLdb database (http://www.cottonqtldb.org) were distributed unevenly across the 26 chromosomes of the cotton genome (**Figure 2C**; Said et al., 2015a). In total, 132 QTLs were linked within chromosome 25, while 15 mapped in chromosome 22 with the least number of QTLs. Chromosome 25 had been dissected for stable QTLs associated with fiber strength and fiber length (Sun et al., 2012; Jamshed et al., 2016).

More than 50% of QTLs for each fiber quality traits showed R^2^ ≥ 10% (**Figure 2D**), implying that genome-wide PV explained by single QTL was comparatively high. These results suggested different mechanisms are involved in the fiber quality traits and showed complex genetic architecture.

### Potential QTLs Mapped for Key Fiber Quality Traits

QTL studies across multiple generations and environments allow the identification of common QTLs for MAS. As mentioned above, fiber length and fiber strength are important economic indicators for fiber quality in cotton. Tan et al. (2015) identified several QTLs for fiber length (*qFL07.1*), fiber strength (*qFS07.1*), fiber micronaire (*qFM07.1*), and fiber elongation (*qFE07.1*) by SSR markers (DPL0757 and BNL1604) in different environments. Wang et al. (2016) utilized immortalized BC populations and identified 22 stable QTLs in many environments and found a potential stable QTL (*qFL-c10-1) in RIL and BCF_1_populations in three environments*. Ali et al. (2018) identified stable QTLs for fiber length and micronaire (*qFL06.1, qFL16.1, qFL21.1*, and *qFM07.1*), which could be further verified by fine mapping and used for candidate gene identification (Ali et al., 2018).

For fiber strength, Zhang et al. (2015) dissected chromosome 25 and identified a comparatively stable QTL for fiber strength (*qFS-chr25-4*) explaining PV of 6.53%–11.83% identified in seven environments. Another, stable QTL (*qFS-C9-1*) on chromosome 09 was mapped within the marker interval of NAU2395 and NAU1092 using an interspecific cross between *G. barbadense* and *G. hirsutum* in different environments and populations (Yang et al., 2016). They focused on fine mapping and cloning genes underlying *qFS-C9-1*, which could provide valuable resources to study the mechanisms for fiber strength. Jamshed et al. (2016) also verified QTL clusters for fiber quality traits on chromosomes c4, c7, c14, and c25 as common QTLs in different environments and populations. Besides, 65 QTLs for fiber length and 15 QTLs for fiber strength were identified by association mapping (Wang et al., 2017). Liu et al. (2018) constructed a high-density genetic map with GWAS analysis of 4,729 SNPs and 122 SSR markers with an average interval of 0.51 cM and harboring a total of 134 QTLs for fiber quality traits in nine environments. A chromosome segment substitution line population developed by crossing between two tetraploid genome species (*G. hirsutum* and *G. barbadense*) detected 103 QTLs associated with fiber quality traits (Li et al., 2019). The introgression of superior alleles of fiber quality from *G. barbadense* contributed to *G. hirsutum* have been linked in five QTL clusters for fiber quality which further associated with significant candidate genes (Gh_A07G1752, Gh_A07G1753, Gh_A07G1754, Gh_A07G1755, and Gh_A07G1756) in fiber development (Chen et al., 2018).

The non-domesticated allotetraploid species (*G. tomentosum*, *G. mustelinum*, and *G. darwinii*) have also been widely utilized in genetic mapping programs for the improvement of fiber quality traits (Keerio et al., 2018). The interspecific population between *G. hirsutum* and *G. mustelinum* (AD_4_) demonstrated potential value in cotton breeding programs for the improvement of fiber quality (Wang et al., 2017). The ILs obtained from crosses between *G. hirsutum* × *G. darwinii* detected 278 polymorphic loci and 13 loci associated with fiber strength among 105 ILs (Wang et al., 2012). Several QTLs were detected across two environments in a RIL population by a genetic map harboring 2618 SNP markers for fiber quality traits and identified 12 QTLs for fiber length and 8 QTLs for fiber strength (Li et al., 2016). A SLAF-seq genetic mapping study conducted with 107 ILs from *G. tomentosum*, identified 30 QTLs linked with fiber quality traits (Keerio et al., 2018). The CottonSNP80K array was utilized for a GWAS study combined with RNA-seq analysis of fiber development stages to predict several candidate genes with differential expression patterns (Dong et al., 2019). Some studies including stable QTLs for fiber quality traits obtained from different mapping techniques have been described in (**Table 2**). The introgression of these stable QTLs into an elite cultivar by marker-assisted BCing will improve fiber traits in cotton.

**Table 2:** List of stable QTLs for fiber-related traits in different studies.

Trait	QTL	Flanking marker	Position	Likelihood of odds score	Var%	Population	Strategies	References
Fiber length (FL)	*qFL21.2*	TM76374-TM76405	109–189	5.1–10.3	2.40–4.00	Recombinant inbred line (RIL), F_2:8_	CottonSNP80K Array	Tan et al., 2018
	*qFL06.1*	Marker3681	35.14	4.00	11.1	RIL, F_2:7_	Specific locus amplified fragment sequencing (SLAF-seq)	Ali et al., 2018
	*qFL16.1*	Marker18806	63.41	2.40	6.6			
	*qFL21.1*	Marker21777	46.14	6.90	18.1			
	*qFL-D03-1*		95	10.44	22.03	F_2:3_	Association mapping	Zhang et al., 2019
	*qFL-D03-2*		94	8.76	16.52	F_2_		Zhang et al., 2019
	*qFL-Chr5-2*	PGML1917-SWU17715		4.8–6.3	14.07–34.00	RIL, backcross population (BC)	Simple sequence repeats (SSR) markers	Ma et al., 2017
	*qFL-Chr5-3*	Gh388-SWU17713		5.39–10.22	10.65–19.63			
	*qFL-LG10–1*	LG10-M5173-LG10-M4596	71.11	8.67	20.7	RIL, F_2:6_	Genotype by sequencing (GBS) genotyping	Fan et al., 2018
	*qFL-C7-1*	DC40182	10.4–12.7	4.27–7.17	3.43–6.98	RIL, F_2:3_	SSR markers	Chen et al., 2018
	*qFL-TX34-A6-1*	Gh591	23.6	3.3–4.8	6.1–9.1	BC_3_F_2:3_	QTL mapping	Feng et al., 2019
Fiber strength (FS)	*qFS-chr01-2*	TM379-TM404	27.41	3.5–5.13	5.3–8.8	RIL, F_6:8_	GWAS	Liu et al., 2018
	*qFS-chr07-2*	DPL0852-DPL0757	69.01	2.66–9.27	5.81–19.47			
	*qFS-chr16-3*	SWU2707-DPL0492	15.61	2.12–2.83	4.28–6.45			
	*qFS-C7-1*	DC40182	10.4–20.3	3.0–19.64	3.0–18.47	F_2:3_	SSR markers	Chen et al., 2018
	*qFS19.1*	TM57229-TM57167	50.938	2.34–3.22	6.1–8.0	RIL, F_2:8_	CottonSNP80K Array	Tan et al., 2018
	*qFS-A09-1*	M993	46.41	7.08	21.61	BC_5_S_5_	SLAF-seq	Keerio et al., 2018
	*qFS-D12_cb*	mk17994_D12-mk17997_D12	65.41	4.6	7.2	F_2:3_	GBS genotyping	Diouf et al., 2018
Fiber maturity (FM)	*qFM-chr07-1*	DPL0852-DPL0757	69.01	2.50–7.44	5.51–24.45	RIL, F_6:8_	GWAS	Liu et al., 2018
	*qFM-C7-1*	DC40182	8.4–21.3	11.41–18.41	10.61-18.80	F_2:3_	SSR markers	Chen et al., 2018
	*qFM24.1*	TM69870-TM69911	119.333	3.20–5.34	7.9–13.5	RIL, F_2:8_	CottonSNP80K Array	Tan et al., 2018
	*qFM07.1*	Marker5551	135.94	6.7	17.4	RIL, F_2:7_	SLAF-seq	Ali et al., 2018
	*qFM-C7-1*	DC40182	8.4–20.3	11.41–18.41	10.61–18.80	F_2:3_	SSR markers	Chen et al., 2018

### Strategy for Fine Mapping of Fiber Quality Traits QTLs

Cotton fiber quality traits are controlled by multiple genes with minor effects and are affected by environmental factors, which take a long period to improve fiber quality through conventional genetic improvement techniques. The DNA markers provide promising means to dissect the mechanisms underlying the traits with complex nature, which allows the breeders to select the region with desirable effects after cycles of MAS (Young and Tanksley 1989). Several studies have mapped fiber QTLs in past decades (Said et al., 2013; Fang et al., 2014; Shang et al., 2015; Tang et al., 2015; Tan et al., 2015; Ma et al., 2017; Ma et al., 2018); these QTLs mapped in large genomic regions that may include hundreds or thousands of genes. Therefore, the target genes with less ‘linkage drag’ can be identified by fine-mapping of QTLs.

#### Studies Involved Fine Mapping of Fiber Quality Traits

Fine mapping of QTLs related to fiber quality traits in marker-assisted breeding relies on narrowing down the genetic basis by producing near isogenic lines (NILs) and ILs. NILs have been constructed with the selection of only one locus from diverse species (Fridman et al., 2000; Brouwer and Clair 2004; Thomson et al., 2006; Szalma et al., 2007; Zhang et al., 2009; Ding et al., 2011; Chen et al., 2012). ILs have been utilized in reducing the QTLs regions from 20 to 3 cM by fine mapping for important traits in tomato, rice, and rape (Paterson et al., 1990). Later, stable QTL for fiber bundle strength was mapped by utilizing two NILs MD52ne and MD90ne, which provided the prospects to study the genetic components involved in cotton fiber strength (Islam et al., 2014).

Su et al. (2013) developed ILs using a marker-assisted breeding approach with TM-1 and fine-mapped fiber strength QTL (*QFS-D11-1*) that exhibited 35.8% PV, into a marker interval of 0.6 cM within flanking markers (NAU2110 and NAU2950). Later, a *G. barbadense* introgressed line, developed by Cao et al. (2015), led to a fine-mapped QTL at same position between the two SSR markers, NAU3735 and NAU845. The intervals of putative QTLs, *qFL-chr.7* for fiber length and *qFS-chr.7* for fiber strength, were decreased to 0.36 and 0.44 cM, respectively (Cao et al., 2015). Because the fiber length and fiber strength are usually positively correlated, there might be evidence of pleiotropy. Furthermore, the integration of Mapping-By-Sequencing technique facilitated the identification of candidate genes in mapped QTLs for fiber bundle strength, short fiber index, and fiber length. The outcome of Mapping-By-Sequencing strategy validated the QTLs and suggested the role of the receptor-like kinases pathway genes in fiber strength (Islam et al., 2016).

#### RNA-Seq Facilitates Fine-Mapping in Identifying Fiber Quality Related Genes

The choice of fine mapping, enhanced by development of transcriptomic datasets and candidate genes identification was supported by the integration of quantitative genetics and transcriptomics. The combination of fine-mapping of QTLs and RNA-seq is a potential strategy for the identification of candidate genes for several complex fiber quality traits. Liu et al. (2016) established a combine approach of fine-mapping and RNA-seq to elucidate the molecular basis of fiber quality by identifying candidate genes underlying a previous QTL near T_1_ locus on chromosome 6. Recently, one QTL was mapped to an interval of 0.28 cM with the flanking markers (HAU2119 and SWU2302), whereas RNA-seq results of ovules at 0 DPA and 5 DPA fibers showed that four genes in this QTL region were expressed differentially and the expression of three genes encoding XTH, ALDH and GPI-anchored protein were significantly linked to fiber elongation (Liu et al., 2016). A potential candidate gene (RLK family protein) for QTL *qFS07.1* was revealed by a comparative approach of RT-qPCR and fine-mapping (Fang et al., 2017). A fiber length QTL (*qFL-chr1*) on chromosome 1 was fine-mapped to a 0.9 cM interval and the candidate genes (GOBAR07705 and GOBAR25992) were associated with fiber length in a BC_4_F_2_ population (Xu et al., 2017). The ILs between *G. hirsutum* and *G. barbadense* were dissected for fiber quality QTL analysis and 13 QTLs were integrated with 235 highly expressed genes during fiber development, and the transcriptome analysis of 10 DPA of these lines led to the identification of eQTLs (expression QTLs) for 125 of these 235 genes (Wang et al., 2019). Among these 235 genes, the *Ghir_D09G014120* and *Ghir_D09G014460* that respectively encoded ubiquitin extension protein and a microtubule-associated protein could be possible candidate genes for fiber strength (Wang et al., 2019). The fuzzless gene (*GaFzl*) was fine mapped to a 70-kb region linked with seven genes in the region, further RNA-seq and re-sequencing analysis helped to narrow down the region up to two candidate genes (Cotton_A_11941 and Cotton_A_11942) with a single-base mutation in the promoter region in a NIL (DPL972) of *G. arboreum* (Feng et al., 2019).

## Fiber Quality Improvement by Mas

The QTLs that have been identified in different populations and in different environments provide predominant evidence of putative loci related with fiber quality traits for MAS. Therefore, high density genetic mapping is a pre-requisite for marker assisted breeding. Once a stable QTL is found to be linked with the desired trait in a certain population, a composite fine-mapping approach will help to dissect the markers close to the target genes. Generally, MAS will be less effective for traits that are governed by many genes such as fiber quality traits. The breeding design and implementation scheme also depend on the efficiency of MAS. Therefore, QTLs that were consistently detected across multi-environments and retained the maximum proportion of PV should be selected for MAS (Flint-Garcia et al., 2003).

Fiber length and strength are important fiber quality traits and plenty of associated QTLs have been identified in previous decades. During the last decade, SSRs have been widely used for MAS for fiber quality in many interspecific population combinations. The stable QTLs for fiber length and strength (*qFL-C7-1*, *qFL-chr.7*, and *QFS-D11-1*) can be utilized in MAS programs to enhance fiber length and fiber strength of commercial cultivars in different populations. QTLs (*qFL19.1*, *qFS03.1*, and *qFM19.1*) identified under multi-environments and different populations have more chance to be a successful MAS program. Furthermore, markers flanking the QTL interval help to identify QTLs with different populations in different environments. Flanking markers of QTLs for fiber length (NAU1085-BNL1694, NAU1085-TMB1618, and NAU3298b-CM067) and fiber strength (BNL2572-BNL1440) could be the best targets to find novel QTLs controlling fiber quality traits in mapping populations (Lacape et al., 2005; Sun et al., 2012; Zhang et al., 2015). Fine-mapping of putative QTLs can also enhance the reliability of MAS and be helpful in developing the best fiber quality cotton cultivars.

Recently, GWAS analyses have been used to identify the key genes that were altered during the cotton fiber evolution. Two genomic loci for *LINT YIELD INCREASING* (GhLYI-A02 and GhLYI-D08) were identified with the mix of GWAS and gene-based association with functional annotation of identified orthologs in *Arabidopsis*, providing the quick method to identify the candidate genes associated with fiber quality (Fang et al., 2017a). Ma et al. (2018) characterized genes related to fiber length (*GhFL1* and *GhFL2*) and fiber strength (Gh_A07G1769), giving targets for genetic improvement to fulfill the demand for fiber quality (Ma et al. 2018b). Cotton fiber quality improvement has occurred under domestication during evolution process and is certainly associated with fiber strength and fiber micronaire genes (MYB, ADF, and CAD) (Wang et al., 2017b). Genome wide analyses of introgression populations by genome re-sequencing and the expression of homologous genes related to fiber quality provide references to produce superior fiber (Fang et al., 2017b).

## Efficiency and Targets for Future Fiber Quality Improvement

Markers assisted selection is one of the widely used breeding strategies to improve traits including cotton fiber quality traits with a multifaceted genetic basis. Several genes associated with fiber development have been reported to function at different stages of fiber development in many articles. In past decades, thousands of QTLs in diploid and tetraploid cotton species have been identified in populations developed by interspecific crosses between parents differing in fiber characteristics. The genomic sequences of cotton provide precious resources to develop high-density SSR or SNP based genetic maps. Establishing linkage between phenotype and genotypic interactions, the identification of stable QTLs lays a basis for fine mapping to dig out the related genes.

With genomic and bioinformatics approaches, it is more feasible to retrieve target QTL regions. The integration of transcriptomic analysis and QTL mapping can reveal more concrete information about fiber development mechanism. These studies should be improved in corresponding cotton genomes and among the populations developed by interspecific crosses. Cotton genomes, DNA markers and transcriptomic studies can play a major role in dissecting the mechanisms underlying fiber development to cultivate superior varieties with improved cotton fiber quality.

## Author Contributions

BI wrote the manuscript. NZ participated in the discussion and the manuscript revision. JK participated in data collection and discussion. JH proposed the idea, instructed the research, revised the manuscript, and provided the work platform. All authors approved the final manuscript.

## Funding

This research was supported by a grant from National Key R & D Program for Crop Breeding (2016YFD0100305) to JH, and a part from Key Cultivating Projects of Scientific and Technological Innovation for Xinjiang Academy of Agricultural Sciences (xjkcpy-001) to JK.

## Conflict of Interest

The authors declare that the research was conducted in the absence of any commercial or financial relationships that could be construed as a potential conflict of interest.

## Abbreviations

QTL, quantitative trait loci; MAS, marker assisted selection; GS, genomic selection; PCW, primary cell wall; SCW, secondary cell wall; TF, transcription factors; DPA, days post anthesis; RAPD, random amplified polymorphic DNA; RFLP, restriction fragment length polymorphism; SSR, simple sequence repeats; ISSR, inter simple sequence repeats; SNP, single nucleotide polymorphism; PV, phenotypic variation; LOD, likelihood of odds score; RIL, recombinant inbred line; ILs, introgression lines; NIL, near isogenic lines; IF2, immortalized F2; MAGIC, multi-parent advanced generation inter-cross; FBS, fiber bundle strength; CISPs, conserved intron scanning primers; TDFs, transcript-derived fragments.
